# Raman spectroscopic detection of carotenoids in cattle skin†

**DOI:** 10.1039/d0ra03147j

**Published:** 2020-06-12

**Authors:** Megha Mehta, Rafea Naffa, Wenkai Zhang, Nicola M. Schreurs, Natalia P. Martin, Rebecca E. Hickson, Mark Waterland, Geoff Holmes

**Affiliations:** NZ Leather and Shoe Research Association (LASRA®) Palmerston North 4472 New Zealand megha.mehta@lasra.co.nz; Animal Science, School of Agriculture and Environment, Massey University Palmerston North New Zealand; School of Fundamental Sciences, Massey University Palmerston North New Zealand

## Abstract

Carotenoids, powerful anti-oxidants, play a significant role in protecting the skin from oxidation and help in balancing the redox status of skin. This study was aimed at investigating cattle skin to identify carotenoids in the lower epidermis (grain) and dermis (corium) layers for classification using Raman spectroscopy which is a powerful technique for the detection of carotenoids in cattle skin due to the strong resonance enhancement with 532 nm laser excitation. The spectral differences identified between these two layers were quantified by the univariate analysis of Raman peak heights and partial least squares (PLS) analysis. We compared the performance of the Raman spectroscopy method with the standard method, high performance liquid chromatography. The univariate analysis results demonstrated that the lower epidermis of the skin has a higher concentration of carotenoid than dermis using the carotenoid Raman peaks at 1151 cm^−1^ and 1518 cm^−1^. The carotenoid Raman intensity was linearly correlated with the total carotenoid concentration determined by standard HPLC methods. Partial Least Squares Regression analysis gives excellent results with *R*^2^ = 0.99. Our results indicate that Raman spectroscopy is a potential tool to determine carotenoids in cattle skin with high precision.

## Abstract 2

The lower epidermis and dermis of cattle skin was investigated for the detection of carotenoids using Raman spectroscopy.

## Graphical abstract

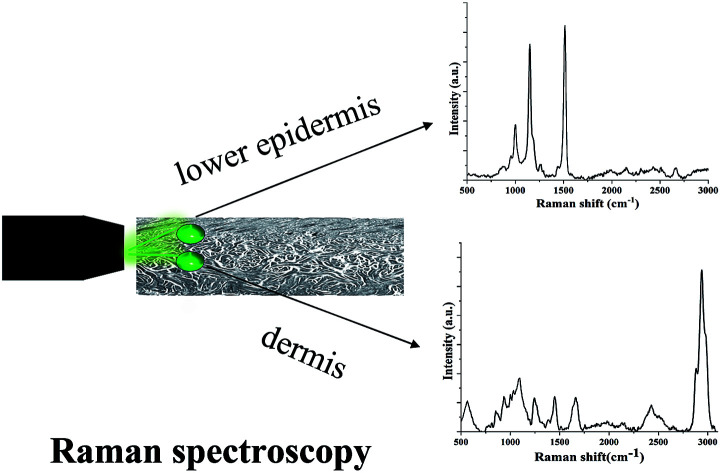

## Introduction

Every year more than a billion animals are processed for meat production. Hides and skins, a by-product of the meat industry, in turn generate returns of over a billion dollars for the global leather industry.^1^ Cattle are the dominant animals across the globe used in the leather industry. The dairy cattle population is at its highest level worldwide, accounting for 65–70% of the world bovine animal population and is used as a source of both meat and dairy products.^2^ Larger countries rely on crops for forage to raise cattle whereas New Zealand (NZ) relies predominantly on pasture.^3^

**Fig. 1:**
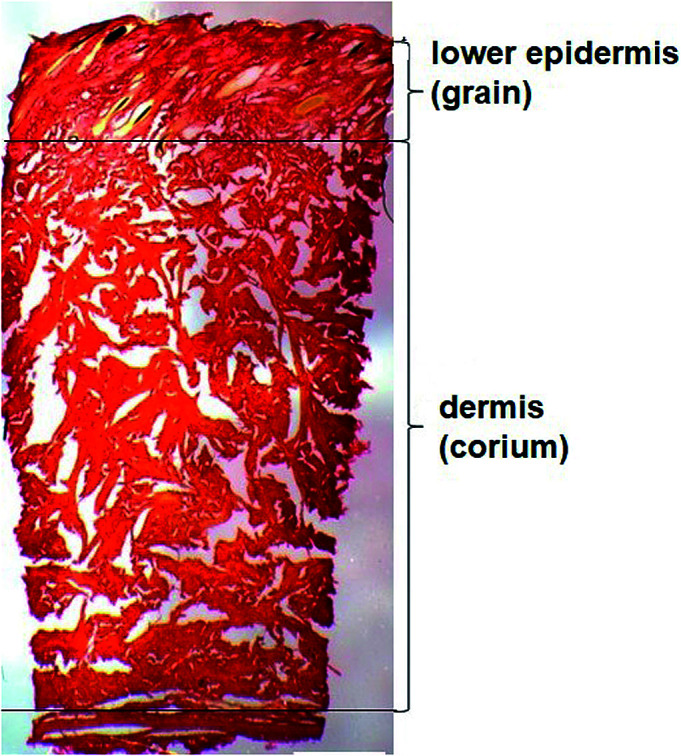
Light microscopy of the skin stained with picrosirius red (scale bar 1000 μm) highlighting the lower epidermis and dermis of cattle skin.

For leather manufacturing, animal skins undergo thorough chemical and mechanical alteration to remove hairs, epidermis and other unwanted materials without damaging the collagen fibres that give the structural firmness to the skin.^4^ Microscopic examination of dairy cattle skin demonstrates two structurally different layers (Fig. 1). The upper layer, originally the outer surface of the skin, has hair embedded in the epidermis which is removed and there is a smooth layer underneath called lower epidermis or grain.^5^ The layer underneath the grain is typically the thicker layer, called the dermis or corium composed of thicker bundles of interwoven collagen fibres.^6^ There is a thin junction found between the two layers which is the grain-corium (terms used in leather industry) boundary zone.^5^ An intact grain layer is required for the leather quality.

The current study is focused on the identification of biomarkers in cattle skin to understand the physiology of skin and then investigate any relation to animal health and animal products. The antioxidant properties of carotenoids protect the skin from the destructive action of free radicals and are responsible for healthy skin in humans and animals.^7^ These antioxidants cannot be synthesised by animals and humans themselves, they must be obtained through the diet.^8,9^ For cattle grazing on fresh pasture, the diet is rich in carotenoids. After digesting these carotenoid rich foods, they get absorbed by body and deposited as body fat in skin, liver, adipose tissue and are found in the blood.^10^ Carotenoid studies have been conducted on human skin for age-related diseases,^11^ or on animals,^12^ birds^13^ or fishes^14^ where the carotenoid pigmentation is important for the commercial market. The epidermis and dermis have the highest concentration of carotenoid and contribute to the animal and human skin color.^15–17^

The dairy cattle used in our study were grazed on pasture and were open to variable environmental conditions, with ultraviolet (UV) exposure a particular concern (we note that cattle are almost exclusively raised in open pasture throughout the year in New Zealand) and that NZ has an “extreme” UV level index.^18^ Carotenoids offer protection against photosensitising compounds^19^ and against facial eczema^20^ which results in severe sunburn. These problems occur in climates with outdoor animals and lots of sunshine, therefore, deserves consideration. The high carotenoid content in cattle skin is making it particularly appropriate for studying carotenoids in the skin,^21^ which can be explained by their diet. Carotenoids offer physiological benefits, and beneficial to immune system function, evidence that carotenoids play a significant role in immune defense remains scant and is generally limited to a handful of studies in mammalian systems.^22–24^

A key challenge to the study is that while the researchers can manipulate the level of internal carotenoids through diet or the physiological need for carotenoids through immune challenges, it has not been possible to directly manipulate the availability of carotenoids within the bodies of animals. Therefore, in this case, there is a clear need for test to assess carotenoids allocation in the skin that can serve as a good model for investigating the link between diet and environmental factors in the larger experiment.

There are several types of carotenoids in human and animal skin including α-carotene, β-carotene, lutein, zeaxanthin, lycopene, lutein, beta-cryptoxanthin but the most abundant carotenoid found among these is β-carotene and lycopene.^17^ The gold standard used for the detection of carotenoids is High Performance Liquid Chromatography (HPLC).^25,26^ The limitation with this technique is the high cost, extensive extraction protocol which destroys the sample, long sample preparation time and cannot be used *in vivo*. Thus, there is a need for an alternative analytical method which requires minimal or no sample preparation, allows the sample recovery after analysis, is quick and has high sensitivity and specificity. Raman spectroscopy is a powerful laser spectroscopic technique that can be used to detect the vibrational energy level of molecules within the sample and gives a spectral ‘fingerprint’ of the individual molecules. It is considered as a valuable tool for the detection and quantification of carotenoids^27,28^ for predicting the skin quality. The positions and intensities of spectral bands^29^ can be assessed for the structural analysis of lower epidermis and dermis layer of skin. Raman spectral analysis can reveal the biochemical information of the skin with minimal amount of sample, delivering fast results, resistant to water interference,^30^ not causing any damage to the sample (with low laser power) and allowing the possibility of *in situ* detection possible. When these carotenoids are excited in the visible wavelength range (532 nm), they behave as an ideal biomarker for Raman analysis because the frequency of the laser radiation that is generating the Raman scattering is in resonance with the frequency of the electronic motions of the carotenoid giving rise to very strong Raman intensities.^31^

The study investigates the chemical variations between lower epidermis and dermis using Raman spectroscopy and identify carotenoids in skin. Firstly, we have acquired the Raman spectra and then examined specific bands using univariate analysis to obtain a perfect classification between lower epidermis and dermis. Logistic regression was performed on univariate data to predict accuracy and precision of the method. Partial least square (PLS) regression was employed on Raman intensities of the carotenoid bands to correlate against the analytical concentrations as determined by HPLC method and the precision and accuracy of carotenoid concentrations in the lower epidermis (grain) and dermis (corium) was determined.

Darvin *et al.*,^32^ demonstrated that carotenoids change with change in lifestyle, diet, seasons with variation in intake of fruits and vegetables, illness, smoking and other different stress factors. This change is due to uncontrolled diet effected by other factors whereas in the current study we have selected a sample data with pasture diet and sampled from the same body site considering no possible diet interferences that can manipulate the analysis. Darvin *et al.* also suggested in his findings that balanced diet rich in fruits and vegetables will increase the concentration of carotenoids, although it is difficult to measure the stress directly. This shows that there is direct correlation between the state of health, diet and level of carotenoids which is the main idea upon which the study is built.

To the best of our knowledge this is the first investigation of carotenoids in pasture-fed animals using Raman spectroscopy. Several studies on detection of carotenoids from plasma, adipose tissue was identified as biomarkers of pasture-feeding,^33,34^ using reflectance spectroscopy but none was done using Raman spectroscopy which brings novelty in the method used.

In addition to animal health, traceability of animal production system is a challenge and an increasingly significant interest for scientists and farmers.^33,35^ There is increasing consumer demand for environmentally sound animal production methods and pasture feeding is associated with green animal production methods. Raman spectroscopy of animal hides provides a convenient method for establishing the traceability of animal products through the supply chain.

## Materials and methods

### Sample preparation

The animals were raised by Massey University Animal Science team at Limestone Downs, near Port Waikato, New Zealand (37°28′S, 174°45′E) with approval from the Massey University Animal Ethics Committee. Complete skins/hides were collected after slaughter, so samples were not obtained on live animals. Skins were collected by New Zealand Leather and Shoe Research Association (LASRA®) from 40 heifers, which were part of the Dairy-Beef Progeny Test funded by Beef and Lamb NZ Genetics. The heifers were sired by either a Hereford (*n* = 22) or Angus (*n* = 18) bull and were from dams that were a mix of Holstein-Friesian crossed with Jersey. After weaning at 100 days of age, the heifers were managed together in a group and continuously grazed on a pasture containing perennial ryegrass, kikuyu and white clover. No other feeds were provided. The 40 heifers were processed for beef on the same day at an average age of 826 days and were an average of 534 kg liveweight at slaughter.

Samples of skin for testing were collected from the same hind-quarter position on the 40 dairy cattle's skin and stored at temperatures 20 °C prior to analysis. The samples were sectioned using a Leica CM1850UV Cryostat to 60 μm thickness. Samples were sectioned laterally in a way that each section of skin includes grain, which is underside epidermis, the dermis and finally a flesh layer at the bottom as depicted in Fig. 1. The samples were prevented from drying by continuously spraying water before the measurement to keep them hydrated. Triplicate sections of each animal were prepared onto microscopes slide for Raman analysis.

### Data acquisition and Raman spectral processing

The samples were analysed using a custom-built Raman microscope based on an inverted IX71 Olympus Microscope A 532 nm excitation laser (with ∼10 mW laser power) was focused onto the sample with a spot size diameter of 1–2 μm using 40× magnification and 0.65 NA objective. A Raman edge filter (12° incident angle) (Iridian Spectral Technologies, Ontario, Canada) directed the excitation into the sample and rejected the Rayleigh scattered light. An additional Raman edge filter (normal incidence) was used to further remove any residual Rayleigh scattering immediately before entering the spectrometer. The Raman scattered light was focussed onto a 50-micron entrance slit of a Teledyne-Princeton Instruments FERGIE spectrometer.

Lower epidermis and dermis were imaged using a light microscope for a hydrated sample of skin mounted on a glass slide.^36^ Triplicates of lateral sections from each cattle skin samples, comprising lower epidermis after shaving of hairs and dermis was used for Raman measurements. Raman spectra were acquired with an exposure time of 5 seconds per frame and 10 frames (each frame was saved separately). In total, 1200 spectra (40 skin samples × 3 sections × 10 frames per spectrum) were collected. The principle of Raman spectrometry is illustrated in Fig. S1.†

Each spectrum was preprocessed with an algorithm written using the SciKit Learn package^37^ in Python 3.7. Baseline correction, background subtraction and average spectra were obtained using the Python algorithm. Then the spectral data was smoothed with five-point Savitzky–Golay smoothing function to smooth spectral noise and normalization was done by dividing each point by the norm of the whole spectrum using Origin 2018b.

To demonstrate the correlation between Raman intensity and the gold standard method, quantitative analysis of the carotenoid concentration was carried out using high performance liquid chromatography (HPLC) (see details below). Calibration curves were created using the peak heights (intensity) of carotenoid bands for univariate analysis. Logistic regression (LR) algorithm 39 was devised to discriminate the samples using the SciKitS Learn package^26^ in Python 3.7.

Partial least squares regression (PLSR) was employed for multivariate analysis. A leave-one-out cross validation method was utilised to assess the performance of the PLSR estimator. PLS is a supervised method that constructs new variables that best describe the relationship between the observed (Raman) data and the predicted variable (carotenoid concentration). Quantification by PLS was performed using Origin 2018b (Origin Lab Corporation, Northampton, MA, USA).

### HPLC system

One cm diameter disks were punched out of cattle skin of the same area with a hole puncher. The disks were lyophilised on a freeze-drier (Labonoco, USA) and weighed. The lyophilised samples were sectioned using a freezing microtome (Leica CM1850 UV, Germany) at 60 μm thickness from the lower epidermis (Fig. S1†). Three sets of 15 sections each were collected from each skin sample representing the first 15 sections as lower epidermis, second 15 sections from lower epidermis–dermis junction and last 15 sections from dermis. All three sets of each skin sample were then weighed into microcentrifuge tubes.

The carotenoid in the sample was extracted with a solvent composed of 20% tetrahydrofuran (BDH Chemicals, New Zealand) in methanol (Fisher Chemical, USA) with 20 mg L^−1^ 2,6-di-*tert*-butyl-4-methylphenol (Roth, Karlsruhe, Germany) as an antioxidant.^38^ Each sample was extracted with 1 mL of solvent, facilitated by vortex and 30 minutes sonication at room temperature. The mixture was centrifuged at 13 000 rpm for 30 minutes then the supernatant containing the carotenoid extract was analysed by HPLC as follow.

The carotenoid content in the extract was separated on an Acclaim C30 column (Thermo Fisher Scientific, USA). The eluent was isocratic 40% isopropanol (Fisher Scientific, United Kingdom) in methanol. Carotenoids were detected by an Ultraviolet/Visible detector (Thermo Scientific, DAD 3000, USA) at a wavelength of 450 nm. β-Carotene (Sigma-Aldrich, USA) dissolved in the extraction solvent and diluted to adequate concentrations was used as the calibration standard. Thirty-six samples from the batch of 40 skin samples were analysed by HPLC for validation with Raman results. The left out 4 samples were not in enough quantity, therefore, not measured by HPLC.

## Results and discussion

### Optical microscopy

Polarised light microscopy was used to examine the lower epidermis and dermis of cattle skin.^36^Fig. 1 shows that the dermis of skin is made up of large collagen fibre bundles. In contrast, the lower epidermis, which is the superficial layer of the epidermis, was made up of smaller and thinner, randomly organised collagen fibres.

### Raman spectra of carotenoids in cattle skin

**Fig. 2:**
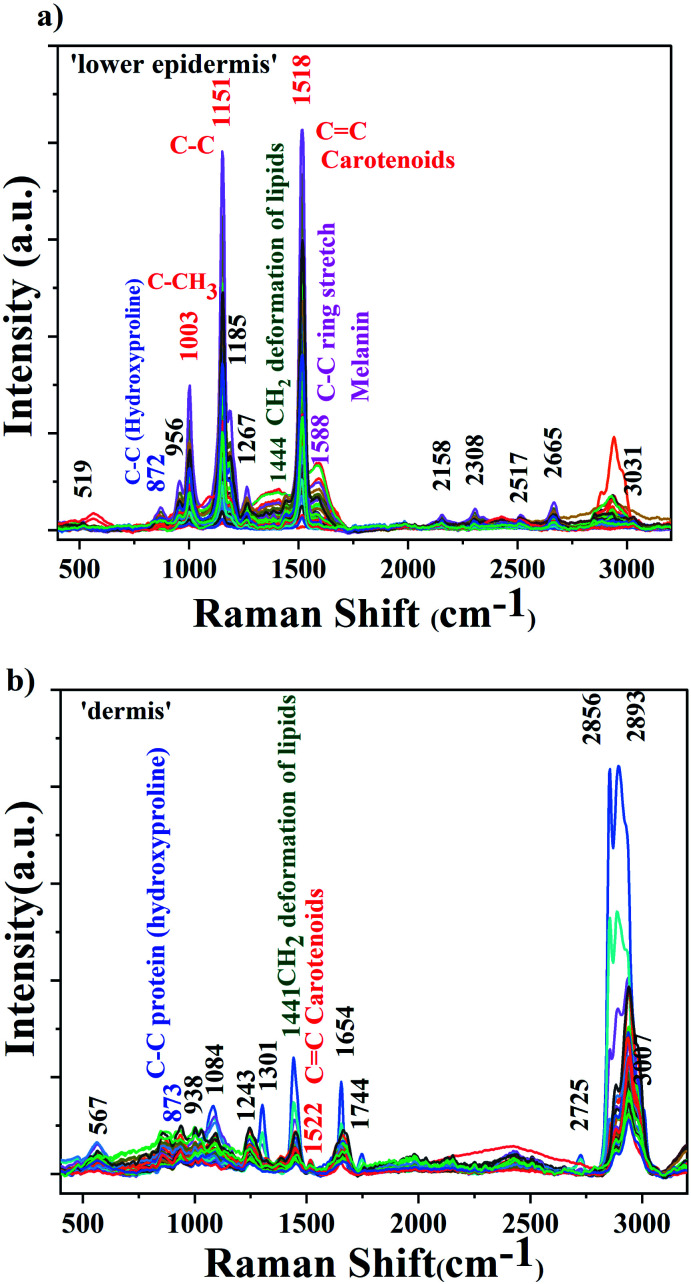
Average Raman spectra of (a) lower epidermis and (b) dermis layer of 40 dairy cattle skins.

The background-corrected Raman spectra of the lower epidermis and dermis layers are shown in Fig. 2.

The raw spectra displayed minimal fluorescence. The three prominent carotenoids peaks identified were at 1003 cm^−1^ due to rocking motions of the methyl group, 1151 cm^−1^ from carbon–carbon single bond stretch vibrations and 1518 cm^−1^ from carbon–carbon double bond stretch vibrations of the conjugated backbone.^39,40^ These results agree with the literature.^31,38^ The band at 1588 cm^−1^ found in lower epidermis of cattle skin contributes to in-plane stretching of aromatic rings of melanin. Raman spectra was acquired under strong resonance conditions with the carotenoids. The resonance effect selectively enhances Raman scattering from the carotenoids due to the strong and narrow carotenoid absorption spectrum. By comparison the melanin enhancement is weaker due to the broad and weak absorption spectrum, so although the vibrational mode frequencies might overlap in some cases, the relative intensity of carotenoid over melanin should be much greater. Therefore, as expected, the carotenoid bands dominate the Raman spectrum and the same is observed.

Several reports claiming no significant difference in the carotenoid status due to variation in the skin tone as reported by Mayne *et al.*^41^

Ermakov *et al.*^42^ also studied optical assessment of skin carotenoid status and demonstrated that large variations in skin carotenoid levels remain detectable independent of the melanin index. The behavior was consistent with the absence of melanin effects on the skin carotenoid levels generated with the different instrument techniques. Statistically significant correlations with melanin levels were therefore absent, or, in other words, there is no indication that subjects with high melanin levels have overestimated carotenoid scores or underestimated carotenoid scores.

For perfect validation of Raman carotenoid results with HPLC without any possible interferents, ultraviolet absorbance scan of carotenoid external standard over a range of wavelengths from 245 nm to 600 nm was obtained which perfectly matched with the extracted carotenoid (Fig. S2†). The obtained absorbance scan does not match with melanin scan^43^ and in excellent agreement with the Flieger *et al.*^44^ report. Based on these considerations, the study was performed for carotenoid determination in cattle skin using Raman spectroscopy and HPLC.

The 1518 cm^−1^ carotenoid band showed the strongest Raman signal in the lower epidermis and was also found with shift of 4 cm^−1^ at 1522 cm^−1^ in dermis. Therefore, 1518 cm^−1^ Raman band was selected to demonstrate the distribution of carotenoids in the lower epidermis and dermis samples of cattle skin. The reason for the slight shift for a few Raman bands could be due to variation in the level of carotenoids in individual skins although they are kept under same environment. These variations depend on the dietary intake rich in carotenoids, age factor and illness in animals.^33^

The lower epidermis shows intense carotenoid bands whereas dermis is mostly dominated by protein and lipid bands.^45^ The lower epidermis is expected to have highest distribution of carotenoids due to the secretion *via* sebaceous or sweat glands onto the skin surface.^17^ Carotenoids penetrate inside the lower epidermis and give the strongest Raman signal in the region. A strong resonance enhancement of carotenoid Raman bands was observed with an acquisition time of just 5 seconds with excellent signal-to-noise that allows sensitive detection of carotenoids in skin.^46^ Triplicate measurements were obtained from each section of the skin sample to ensure reproducibility and high accuracy of results.

A table including all the Raman peaks observed in the lower epidermis and dermis is given in the ESI as Table S1.†

### Distribution of carotenoids in lower epidermis and dermis of cattle skin

The distribution of carotenoids and other components in skin, measured by Raman spectroscopy, depends strongly on the layer examined.^47^ Marker bands at 872 cm^−1^ for protein (hydroxyproline), 1441 cm^−1^ for CH_2_ deformation of lipids and 1518–1522 cm^−1^ for carotenoids due to C–C & conjugated C=C band stretch^48,49^ are used for demonstrating the distribution of various biological components in lower epidermis and dermis. To illustrate the distribution,^50^ samples were reduced to four sets averaging ten skin samples for Raman analysis. Peak height (intensity) of above stated Raman bands was used to measure the distribution in both layers.

**Fig. 3:**
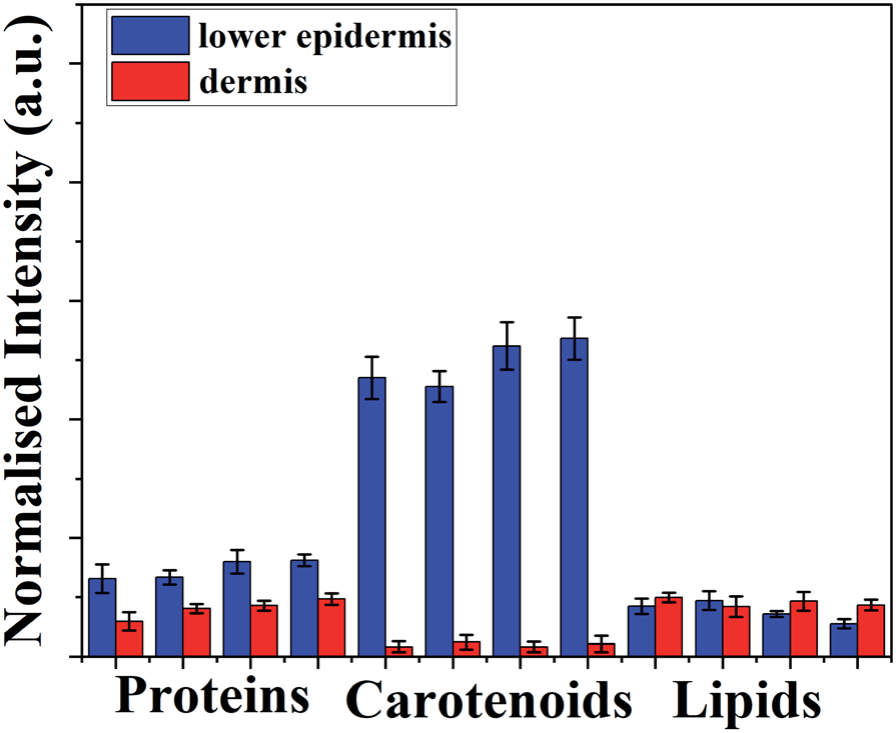
Average peak intensity ± standard deviation for proteins, carotenoids and lipids in lower epidermis and dermis of cattle skin measured with Raman spectroscopy. Error bars are the standard deviation for each averaged sample set.

Fig. 3 shows there is a minimal increase in protein distribution from the lower epidermis to the dermis whereas lipid content in dermis is higher than found in lower epidermis, but carotenoids shows the highest difference between two layers of the skin – eight-fold higher than lipids and four-fold higher than the protein distribution (Table S2†).

The concentration of total carotenoids in the extracted phase of^36^ skin samples (as mentioned in the extraction protocol) was measured and analysed spectroscopically.

No carotenoid was found in the dermis of sample number 3, 4 and 10, which suggests that most of carotenoids are deposited in the outer layer of the skin. HPLC only measures the total carotenoids and does not demonstrate the distribution of carotenoid at the lower epidermis in different cattle skins.

**Fig. 4:**
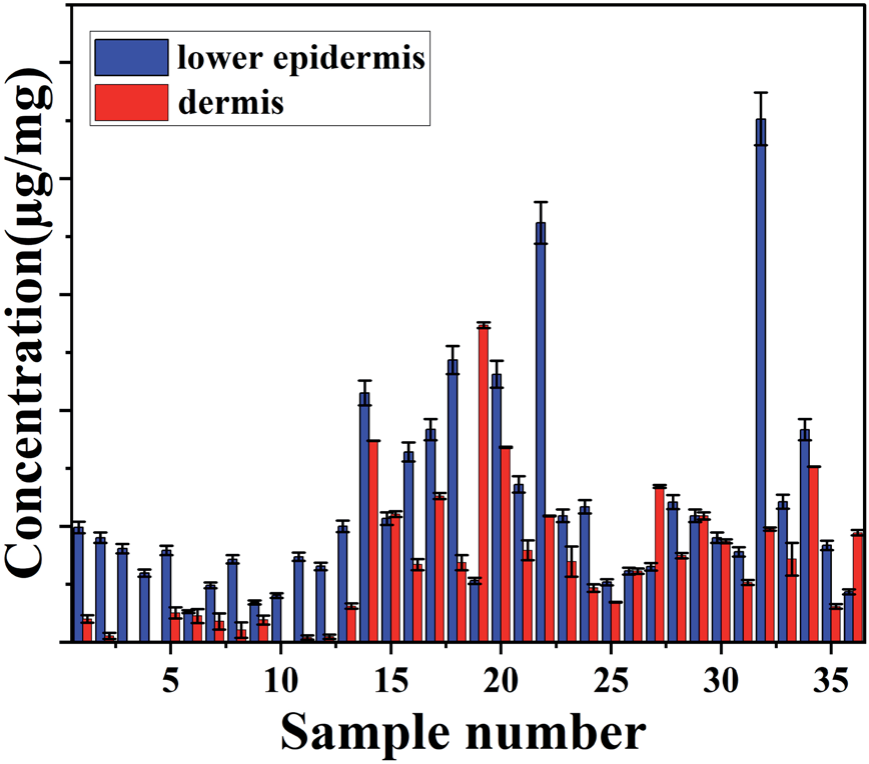
Concentration of carotenoids (μg mg^−1^) in lower epidermis and dermis of cattle skin measured with HPLC.

Both measurement methods showed a good agreement with gradual decrease in carotenoids from lower epidermis to dermis of cattle skin (Fig. 4).

### Quantitative determination of carotenoid concentration by Raman spectroscopy

#### Univariate analysis

Results obtained from Raman spectroscopy and HPLC indicates the higher concentration of carotenoids in lower epidermis. Therefore, univariate and multivariate analysis was performed on the lower epidermis for validation of Raman scattering method.

**Fig. 5:**
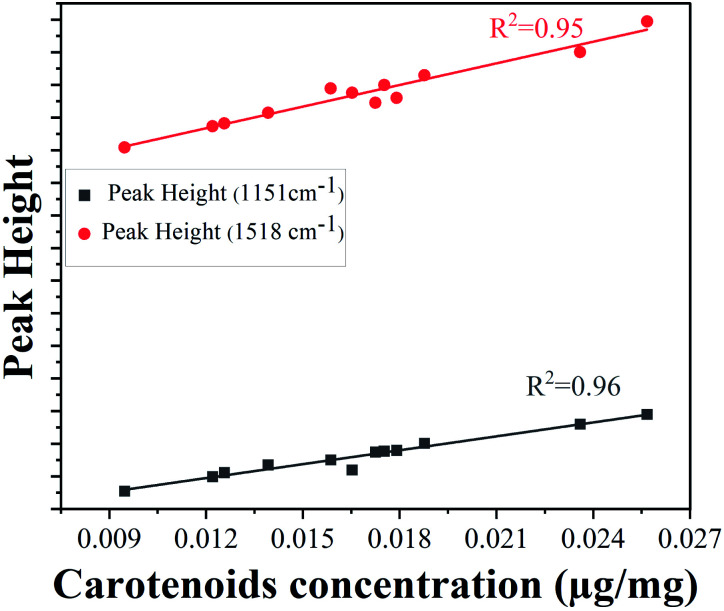
Calibration curves using peak heights 1151 cm^−1^ (bottom) and 1521 cm^−1^ (top) and carotenoid concentration (μg mg^−1^).

The lower epidermis has two strongest peaks at 1151 cm^−1^ and 1518 cm^−1^. The peak intensities of both Raman bands were used for validation with concentration of carotenoids obtained from HPLC results to ensure good reproducibility. The calibration curves using the heights of the carotenoid Raman peaks at 1151 cm^−1^ and 1518 cm^−1^ are linearly correlated (Fig. 5) with total carotenoid concentration in the lower epidermis determined by HPLC method (with correlation coefficients 0.96 and 0.95, respectively), also reported by various other groups.^51–53^

#### Logistic regression

Quantitative classification of lower epidermis and dermis of skin involves a continuous independent variable (peak heights) and a binary dependent variable (lower epidermis *vs.* dermis), therefore a logistic regression (LR) algorithm^54^ was employed.

A confusion matrix was generated from the output that describes the performance of classification. It summarises correct and incorrect spectra classification. It is useful for two-class classification and in measuring recall, precision and accuracy.^54–56^ The confusion matrix for Raman data obtained is presented in Table 1.

**Table 1:** Confusion matrix for Raman univariate analysis

Actual values	Classifier prediction	
	Lower epidermis	Dermis
Lower epidermis	4	0
Dermis	0	4

The data presented in Table 1 shows the perfect classification of 4/4 from all four-average set of lower epidermis and dermis.

Accuracy, precision and recall are of importance where:
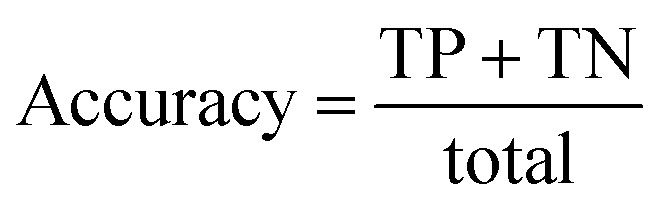

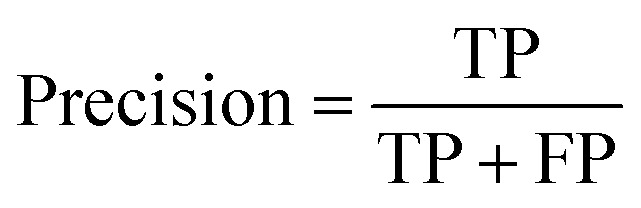

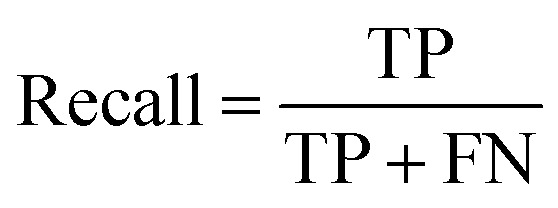
where TP = true positive, TN = true negative, FP = false positive, and FN = false negative with DA arbitrarily set as true and OSP set as false.

The accuracy, precision and recall score for carotenoids in lower epidermis and dermis is 1.0 (100%). Although univariate analysis is quite useful, it might be possible to still obtain a useful prediction from Raman spectra by using a multivariate analysis to reveal the differences, especially when there is a large dataset.

### Comparison of univariate analysis with multivariate analysis

**Fig. 6:**
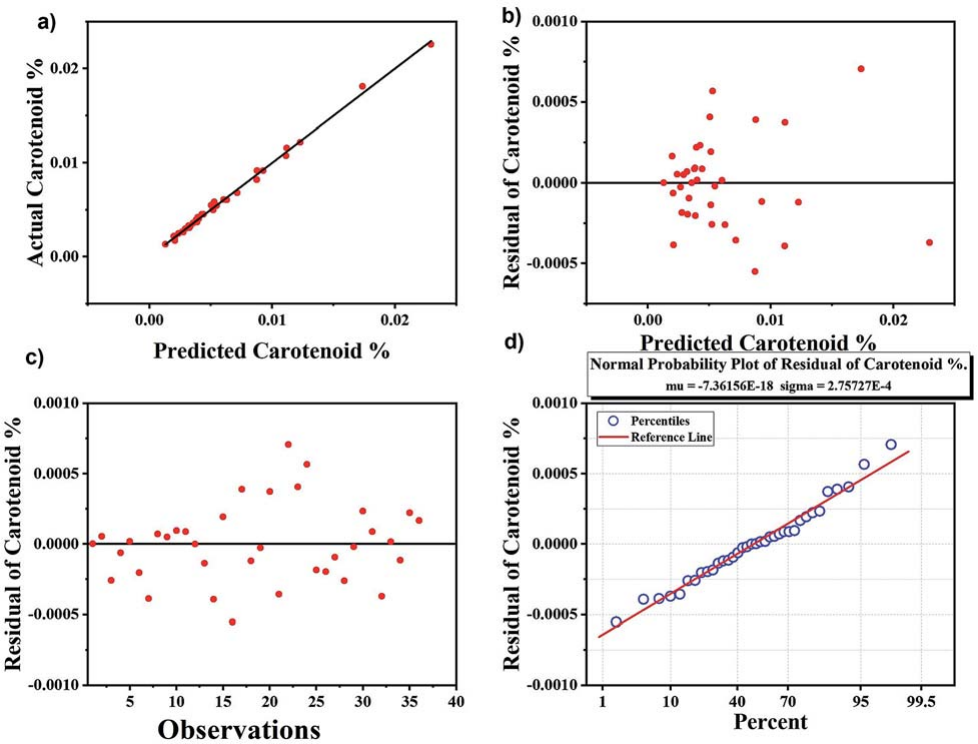
Results of calibration set of carotenoids using partial least square regression analysis. (a) Predicted carotenoid *vs.* actual carotenoids, (b) predicted carotenoids *vs.* residuals, (c) number of observations *vs.* residual carotenoids, (d) normal probability plot indicating the percentile variance with respect to residual carotenoids.

The results obtained from univariate analysis were compared with multivariate analysis using PLS regression, which is another approach for quantification.^57^ The results obtained are shown in Fig. 6. Good precision was obtained with PLS analysis with *R*^2^ as 0.98 whereas *R*^2^ value obtained using peak height analysis for univariate analysis was slightly lower than that of the multivariate analysis. All samples are accurately classified using PLS model. The advantage of PLS method is that it includes several predictive variables even when the sample size is small. The residual plots in Fig. 6 are used to evaluate the quality of the prediction model. The predicted *vs.* actual carotenoid values fits very well with each other (Fig. 6a) and the residuals graph (Fig. 6b and c) also demonstrate that there is no drift in the process. The normal probability plot of residual results also falls in a line represents that variance is normally distributed (Fig. 6d).^58^

The PLS regression analysis perform better than the univariate analysis of peak height but both methods provide the accuracy for the quantification of carotenoids. Therefore, it is believed that employing both univariate and multivariate analysis for Raman spectroscopy allowed us to quantify the method with uniform results of large sample sizes.

As a negative control, the HPLC run samples were again analysed by Raman spectroscopy to detect if there are any carotenoids left in the samples, but no trace of carotenoids was obtained indicating that carotenoids were fully extracted from the samples, identified using HPLC and validated finally by Raman spectroscopy.

## Conclusions

The current paper demonstrates greater concentration of carotenoids in the lower epidermis of cattle skin than in the dermis. These skin carotenoids are used as biomarker for Raman analysis. Univariate analysis and PLS methods were used for cross-validating Raman results with HPLC data. Univariate analysis results showed slightly less accuracy as compared to PLSR method. Our results demonstrate that Raman spectroscopy is an accurate and precise for carotenoid quantification method and can be used to monitor the aspects of animal health.

To understand the impact of oxidative status of the cattle with respect to diet and age can be further investigated in future studies. Investigating the skins based on carotenoids can open several connections of animal to diet, age and exposure to skin diseases, which is very important for healthy skin.

## Conflicts of interest

There are no conflicts to declare.

## Supplementary Material

RA-010-D0RA03147J-s001
