# Supplementary material for: Raman spectroscopic detection of carotenoids in cattle skin
Source: RSC Adv. 2020 Jun 12;10(38):22758–65. doi: 10.1039/d0ra03147j (PMC9054613; doi:10.1039/d0ra03147j)
Supplement: RA-010-D0RA03147J-s001 [file RA-010-D0RA03147J-s001.pdf]

Electronic Supplementary Material (ESI) for RSC Advances.
This journal is © The Royal Society of Chemistry 2020

Supporting Information

Raman spectroscopic detection of carotenoids in cattle skin

*Correspondence to Megha Mehta: (Email: megha.mehta@lasra.co.nz)

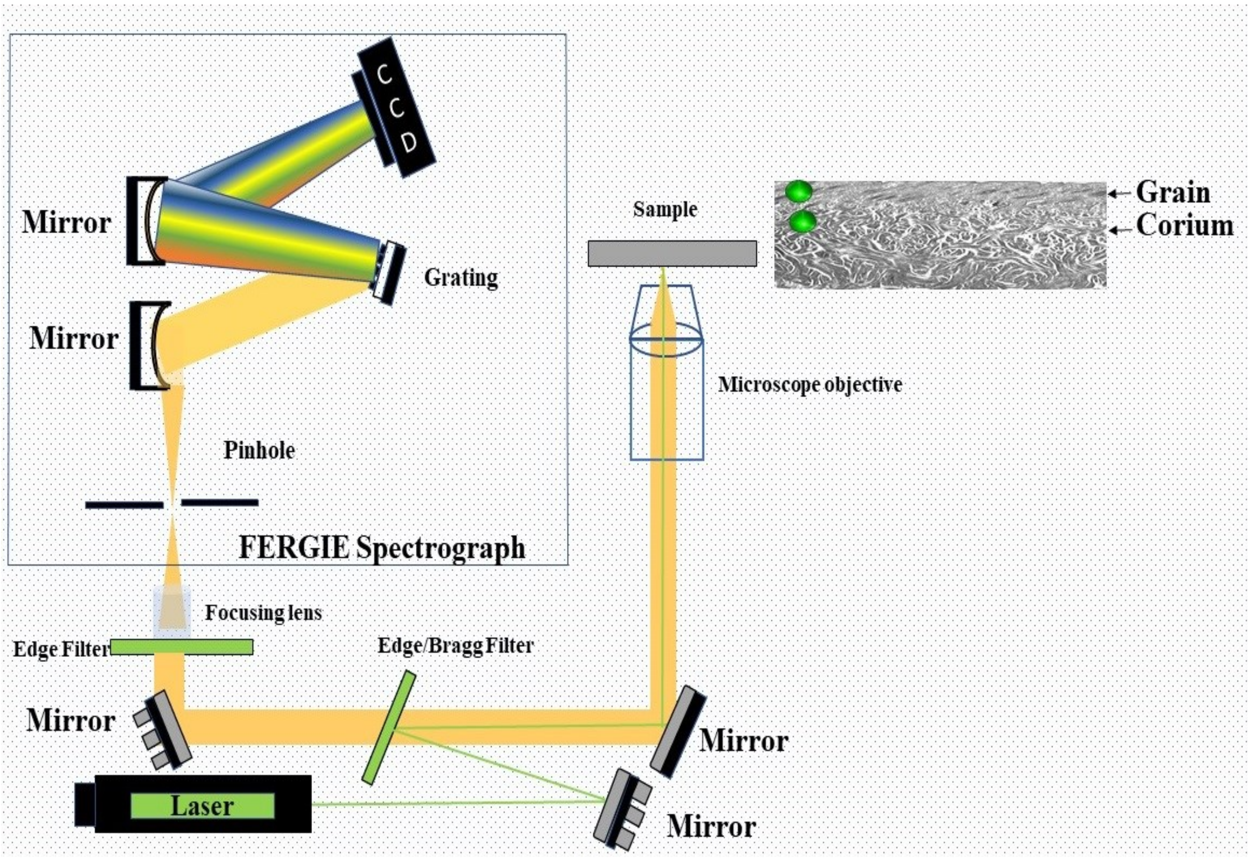

Fig. S1 Illustration of Raman spectroscopy hardware and setup with hide sample.

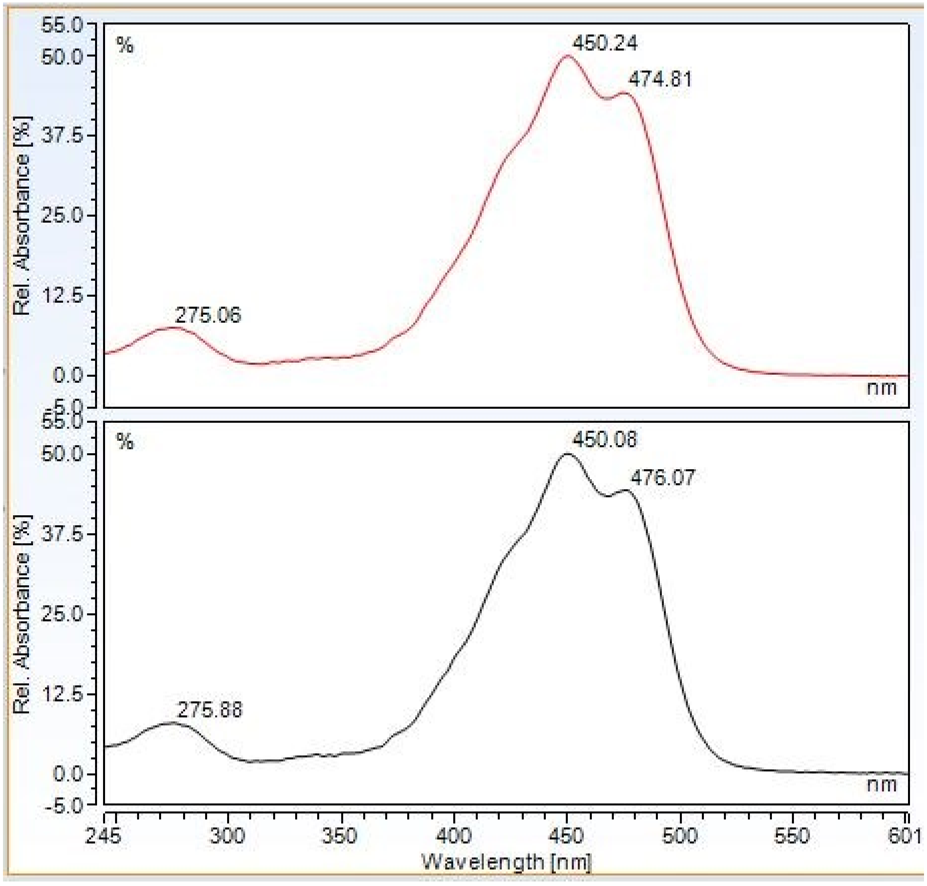

Fig. S2 UV scan of carotenoid external standard (top) and carotenoid extracted from the sample (bottom).

Table S1. Tentative assignments of Raman bands (grain and corium layer of cattle hide)

Position of character peak (cm^{-1})	Biochemical Assignments	Biomolecular Assignments	Skin layer
**515**	S-S stretch	Keratin	**GRAIN**
**567**	N-H stretch	Cytosine or Guanine	**CORIUM**
**872-873**	*ν* C-C Hydroxyproline	Protein (collagen)	**GRAIN, CORIUM**
**938**	*ν* C-C Proline and valine (α-helix)	Protein (collagen)	**CORIUM**
**956**	δCH_3 (deformed)	Lipid, protein	**GRAIN**
**1001–1004**	*ν* $C\text{-}CH_3$ ring breathing	Phenylalanine, Carotenoids	**GRAIN**
**1084–1095**	*ν* -O-P-O_{-}, *ν* C-C phospholipid	Nucleic acid (DNA, RNA)	**CORIUM**
**1151.00**	*ν* C-C	Carotenoids	**GRAIN**

**1243**	Amino compound III (β fold)		**CORIUM**
**1267**	Amino compound III (α-helix)		**GRAIN**
**1288–1304**	Lipid CH_2 bending vibration and bending vibration CH_2CH_3		**CORIUM**
**1351**	Carbon particle		**GRAIN**
**1388**	CH_3 band		**GRAIN**
**1441-1449**	δCH_2 (bending) proteins and lipids	Protein, Lipid	**GRAIN, CORIUM**
**1518-1522**	*v* C=C	Carotenoids	**GRAIN, CORIUM**
**1588**	*v* C-C aromatic ring	Melanin	**GRAIN**
**1654,1657**	Amino compounds I, α helix	Protein	**CORIUM**
**1744**	C=O stretch	Carbonyl feature of lipid spectra, Ester group	**CORIUM**

**2154**	*v* C≡N		**GRAIN**
**2305-3100**	*v* C-H	CH_3 symmetric stretch of lipids	**GRAIN, CORIUM**

v: stretching vibration, *va*s: asymmetric stretching vibration, *v*s: symmetric stretching vibration, δ: bending, deformed, swing (relative peak intensity = the peak intensity/average intensity of the full spectrum).

Table S2: Distribution of biochemical components in Grain and Corium Layers of hide.

**Number**	**Normalised Intensity**					
	**Lipids**		**Proteins**		**Carotenoids**	
	**Grain**	**Corium**	**Grain**	**Corium**	**Grain**	**Corium**
1	0.042	0.049	0.065	0.029	0.23	0.008
2	0.047	0.041	0.066	0.040	0.22	0.01
3	0.035	0.046	0.079	0.042	0.26	0.007
4	0.027	0.043	0.08	0.048	0.26	0.010
**Average**	0.0380	0.045	0.073	0.040	0.24	0.009
**SD**	0.0085	0.0035	0.008	0.0079	0.019	0.0019

Table S3: HPLC results for correlation with Raman results.

Sample	Grain layer β-carotene concentration (μg/mg)
37	0.04957
59	0.04513
42	0.04042
55	0.02982
57	0.03962
66	0.01317
31	0.02451
26	0.03579
44	0.01710
40	0.02013
43	0.03690
54	0.03276
27	0.0501
28	0.1075
29	0.0534
30	0.082
32	0.0917
33	0.1217
34	0.0265
35	0.1156
36	0.0681
52	0.1808
39	0.0546
41	0.0585
46	0.0259
50	0.0307
51	0.0326
53	0.0605
56	0.0546
58	0.0451
48	0.039
61	0.2257
62	0.0607
63	0.0916
64	0.0418
67	0.0217
